# Symptoms and biomarkers associated with undiagnosed celiac seropositivity

**DOI:** 10.1186/s12876-021-01667-y

**Published:** 2021-02-27

**Authors:** Line Lund Kårhus, Janne Petersen, Katja Biering Leth-Møller, Line Tang Møllehave, Anja Lykke Madsen, Betina Heinsbæk Thuesen, Peter Schwarz, Jüri J. Rumessen, Allan Linneberg

**Affiliations:** 1 Center for Clinical Research and Prevention, Bispebjerg and Frederiksberg Hospital, Hovedvejen, Entrance 5, Nordre Fasanvej 57, 2000 Frederiksberg, Copenhagen, Denmark; 2 Section of Biostatistics, Department of Public Health, University of Copenhagen, Copenhagen, Denmark; 3 The Copenhagen City Heart Study, Bispebjerg and Frederiksberg Hospital, Copenhagen, Denmark; 4 Department of Endocrinology and Diabetes and Bone-Metabolic Research Unit, Rigshospitalet, Copenhagen, Denmark; 5 Department of Clinical Medicine, Faculty of Health and Medical Sciences, University of Copenhagen, Copenhagen, Denmark; 6 Q&D-Research Unit and Department of Gastroenterology, Herlev and Gentofte Hospital, University of Copenhagen, Copenhagen, Denmark

**Keywords:** Antibodies, Celiac disease, Epidemiology, Screening, Symptoms, Biomarkers

## Abstract

**Background:**

Studies have indicated that underdiagnosis and diagnostic delay are common in celiac disease. Therefore, it is important to increase our knowledge of what symptoms and biomarkers could identify undiagnosed cases of celiac disease.

**Methods:**

We screened for celiac disease antibodies in stored blood samples from 16,776 participants in eight population-based studies examined during 1976–2012. Undiagnosed celiac seropositivity was defined as celiac disease antibody positivity (IgG-deamidated gliadin peptide above 10.0 U/mL and/or IgA-tissue transglutaminase (TTG) or IgG-TTG above 7.0 U/mL) without a known diagnosis of celiac disease in the National Patient Register. In all studies general health symptoms were recorded by participant-completed questionnaire, including self-perceived health, tiredness, headache and gastrointestinal symptoms. Furthermore, blood samples were drawn for analyses of biomarkers e.g. hemoglobin, blood glucose, cholesterol, liver parameters and vitamins. The participants with undiagnosed celiac seropositivity were matched by sex, age and study with four controls among the celiac disease antibody negative participants.

**Results:**

We excluded, five participants with known celiac disease, resulting in a population of 16,771 participants. In this population 1% (169/16,771) had undiagnosed celiac seropositivity. There were no statistically significant differences in symptoms between cases and controls. Undiagnosed celiac seropositivity was associated with low blood cholesterol (< 5 mmol/L) and low hemoglobin (< 7.3 mmol/L for women and < 8.3 mmol/L for men).

**Conclusion:**

In this general population study, undiagnosed cases of celiac seropositivity did not have more symptoms than controls, confirming the diagnostic difficulties of celiac disease and the low prognostic value of symptoms for a diagnosis of celiac disease. Furthermore, decreased levels of cholesterol and/or hemoglobin in the blood were associated with undiagnosed celiac seropositivity.

**Supplementary Information:**

The online version contains supplementary material available at 10.1186/s12876-021-01667-y.

## Background

Celiac disease (CD) is a lifelong autoimmune disease caused by an abnormal immune response triggered by the ingestion of gluten-containing grains (wheat, rye and barley) in genetically susceptible individuals [1]. CD is a systemic disease occurring at every age affecting around 1% of the population [2]. However, many cases of CD remain undiagnosed [1, 3–8]. CD primarily affects the small intestine, but the clinical manifestations are broad. The treatment of CD is life-long gluten-free diet. A small intestinal biopsy, and detection of villus atrophy and inflammation, has been gold standard for the diagnosis of CD. However, detection of CD specific antibodies, mainly immunoglobulin (Ig) A against tissue transglutaminase (TTG), the autoantigen in CD, has become increasingly important in the diagnostic process and screening for CD [1, 9]. Screening for CD among individuals without classical symptoms of CD or in the general population remains a controversial issue, e.g. because many screen-detected cases have few or no symptoms, and little is known about the prognosis of undiagnosed CD. However, screening might be important as a Swedish study found the mean delay to diagnosis was 10 years from the first symptoms and 6 years from the first doctor visit [10]. Additionally, there is evidence to suggest that asymptomatic patients with serological biomarkers for CD may also benefit from a gluten-free diet [11].

In Denmark, the prevalence of diagnosed CD is lower than in other European countries, despite recent increases in prevalence of CD recorded in national registries [12, 13]. Furthermore, we have previously shown that CD is markedly underdiagnosed in the general population: the screening-prevalence was ten times the registered prevalence [14]. Additionally, we found no differences in symptoms before screening among participants with and without screen-detected CD [15], in line with other studies [16, 17]. This illustrates the difficulty of identifying CD by symptoms. In the present study we used the population earlier described in Kårhus et al. [18] with data from eight population-based cohort studies comprising 16,776 participants examined during 1976–2012. We aimed to investigate whether participants with undiagnosed CD, as defined by CD antibody positivity without a known diagnosis of CD, had more symptoms or affected biomarkers compared with CD antibody negative participants.

## Methods

**Fig. 1:**
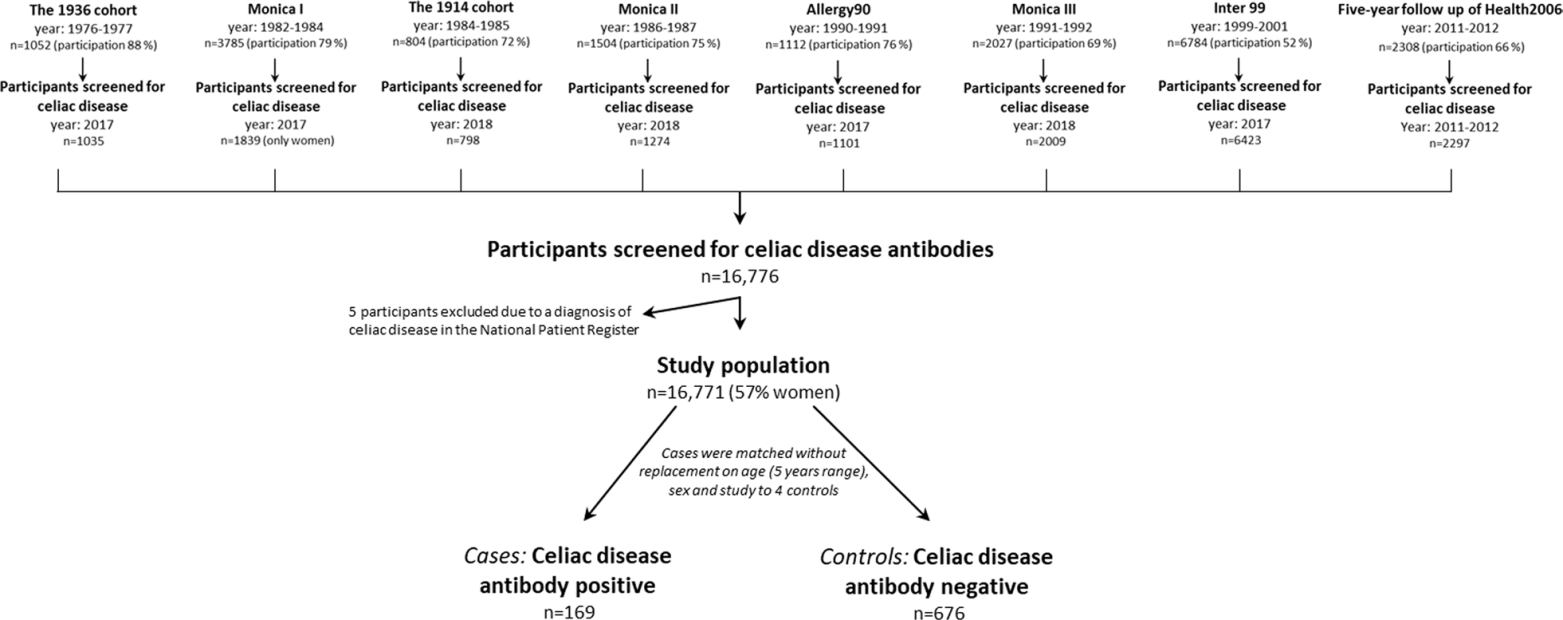
Overview of the studies included, and formation and flow of the study population

As described earlier [18], we used data from eight population-based studies carried out during 1976–2012 at the Center for Clinical Research and Prevention; The 5-year follow-up of The Health 2006 study [14, 15], The Inter99 study [19, 20], The 1936-cohort study [21], The Monica-I study [22], The Monica-II study [23], The Monica-III study [22, 23], The 1914-cohort study [24, 25], and The Allergy 90 study [26, 27]. In all studies a random sample, or an age-stratified random sample, was drawn from persons living in the Western part of Copenhagen, Denmark. Participants were invited to a health examination including self-administered questionnaires and blood sampling. Figure 1 gives an overview of the studies included and formation and flow of the study-population. More information about the studies is included in Additional file 1: Additional Material*.* The screening for CD has been described elsewhere [18]. Briefly*,* 16,776 participants with available serum were screened for CD antibodies by the EliA™ Celikey® TTG anti-IgA and anti-IgG assay, and DGP anti-IgG assays, and 169 participants were identified with undiagnosed celiac seropositivity (Table 1).The measurements were performed at Thermo Fisher Scientific, ImmunoDiagnostics, Allerød, Denmark.

**Table 1:** General characteristics of the cases and controls

	Cases: Celiac disease antibody positive participants^a^	Controls	*p* value ^b^
Number of participants	169	676	
	% (n/total N)	% (n/total N)	
Sex^c^			NA^c^
Male	49.7 (84/169)	49.7 (336/676)	
Female	50.3 (85/169)	50.3 (340/676)	
Education (vocational training)			0.736*
Yes	77.3 (126/163)	78.5 (519/661)	
No	22.7 (37/163)	21.5 (142/163)	
Smoking			0.143*
Current smoker	36.5 (61/167)	41.8 (281/673)	
Past smoker	24.0 (40/167)	26.8 (180/673)	
Never smoker	39.5 (66/167)	31.5 (212/673)	
	Mean (95% CI)	Mean (95% CI)	
Age at examination^c^	49.0 (47.3–50.7)	48.7 (47.9–49.6)	0.759^c‡^
Body mass index (BMI)	26.2 (25.5–26.9)	25.8 (25.5–26.2)	0.353^‡^
	Median (5%/95%)	Median (5%/95%)	
Alcohol consumption: Units per week	5.0 (0.0/33.0)	6.0 (0.0/31.0)	0.198^†^

*Chi-Square test; ^†^Wilcoxon Two-sample test; ^‡^Independent samples *t* test

^a^The participants were screened for celiac disease antibodies by EliA™ Celikey® tissue transglutaminase (TTG) anti-IgA and anti-IgG assays and deamidated gliadin peptide (DGP) anti-IgG assays. Celiac disease antibody positive were defined as IgG-DGP ≥ 10.0 U/mL and/or IgA-TTG/IgG-TTG ≥ 7.0 U/mL. 5 of the 16,776 participants had a diagnosis of celiac disease in the national patient register, they were all celiac disease antibody negative and were excluded before matching

^b^*p* value between antibody negative and antibody positive (case:controls)

^c^Cases were matched without replacement on age (5 years range), sex and study to 4 controls among the antibody negative participans

### Register data

All the identified participants were linked on an individual level to the National Patient Register by use of the unique personal identification number [28]. The National Patient Register holds information on diagnose codes (international classification of disease (ICD)-8 and ICD-10) registered from 1977 [29], for this study we used information on registered CD, i.e. diagnosis codes for CD (ICD-10: K90.0 and ICD-8: 269.00), in order to exclude individuals with a known diagnosis of CD.

### Symptoms

In all studies general health symptoms were recorded by participant-completed questionnaire, including self-perceived health, tiredness, headache and gastrointestinal symptoms. The questionnaires vary through the studies and therefore the wording was not the same in all eight cohorts and some data-harmonization was done, mostly questionnaire answers with more than two answer-categories were dichotomized. The numbers of answers for each symptom in the different cohorts are shown in Additional file 1: Table 1.

### Biomarkers

All studies included blood samples and we examined levels of blood lipids, blood glucose, vitamin D and B12, markers of liver function and anemia as these biomarkers have been shown associated with diagnosed CD. Quartiles for each biomarker were calculated per each study and reference levels for the clinical cut-offs were chosen with reference to guidelines [30]. The methods for biomarker determination and the number of available biomarkers varied between the studies. For the chosen biomarkers in this study the Allergy 90 study had no biomarker results, while the other cohorts had one or more measurements. The numbers and presence of biomarkers for each symptom in the different cohorts are shown in Additional file 1: Table 2.

### Statistics

Cases, the antibody positive participants, were matched without replacement by age (5 years range), sex and study to four controls, antibody negative participants. The 169 participants with undiagnosed celiac seropositivity were matched with four antibody negative participants, giving 676 controls. We compared the cases and controls on general characteristics (Table 1) and found that the groups were similar also on education, body mass index (BMI), smoking and alcohol consumption in addition to the matching criteria sex and age.

Conditional logistic regression was performed in comparisons of cases and controls, to account for homogeneity among matched participants. General characteristics were compared between cases and controls using; Wilcoxon test and Independent samples *t* test for continuous variables; and Chi-Square test for categorical data. Complete case analyses were performed thus the number of observations differs due to missing data. Confidence intervals (CIs) not including 1.0 and *p* values < 0.05 (two-sided) were considered as significant. Statistical analyses were performed with software package SAS 9.4 and SAS Enterprise Guide 7.1 (SAS Institute, NC, USA).

## Results

A total of 16,776 individuals was screened for CD antibodies. Among these, five participants had a diagnosis of CD in The National Patient Register and were therefore excluded, resulting in a study population of 16,771 individuals; 9515 women and 7256 men. The five participants with a diagnosis of CD in the register were all CD antibody negative. The prevalence of undiagnosed celiac seropositivity, defined by CD antibody positivity (IgG-DGP ≥ 10.0 U/mL and/or IgA-TTG or IgG-TTG ≥ 7.0 U/mL) without a known diagnosis of CD, was 1% (169/16,771). The 169 participants with undiagnosed celiac seropositivity were matched with four antibody negative participants, giving 676 controls.

Table 2 shows the comparison of symptoms between cases and controls, both for general symptoms and abdominal symptoms; there were no significant differences between the two groups. However, there was a tendency of more tiredness (29.9% vs. 26.9%) and headache (54.4% vs. 51.1%) among the cases. While, for the abdominal symptoms there was no clear tendency, some symptoms such as bloating, reflux and nausea were more frequent among the cases, but the cases had lower frequency of abdominal pain.

**Table 2:** Comparison of self-reported symptoms^a^ between cases and controls

	Cases: Celiac disease antibody positive participants^b^	Controls	OR (95% CI)^c^
Number of participants	169	676	
	% (n/total N)	% (n/total N)	
Tiredness			
Yes	29.9 (50/167)	26.9 (180/670)	1.14 (0.79–1.66)
No	70.1 (117/167)	73.1 (490/670)	Ref
Self-perceived health			
Very good/excellent	26.2 (38/145)	30.1 (178/592)	Ref
Good	66.2 (96/145)	57.6 (341/592)	1.37 (0.90–2.10)
Fair/poor	7.6 (11/145)	12.3 (73/592)	0.73 (0.35–1.52)
Headache			
Yes	54.4 (81/149)	51.1 (314/615)	1.32 (0.80–2.18)
No	45.6 (68/149)	48.9 (301/615)	Ref
Abdominal pain			
Yes	24.9 (41/165)	27.1 (181/669)	0.92 (0.62–1.37)
No	75.2 (124/165)	72.9 (488/669)	Ref
Bloating			
Yes	65.8 (77/117)	64.0 (301/470)	1.12 (0.72–1.74)
No	34.2 (40/117)	36.0 (169/470)	Ref
Reflux			
Yes	44.3 (31/70)	39.0 (112/287)	1.26 (0.73–2.17)
No	55.7 (39/70)	61.0 (175/287)	Ref
Rumbling			
Yes	42.0 (66/157)	42.3 (294/622)	0.82 (0.57–1.18)
No	58.0 (91/157)	52.7 (328/622)	Ref
Alternating stool			
Yes	68.0 (106/156)	67.6 (421/623)	1.02 (0.69–1.51)
No	32.1 (50/156)	32.4 (202/623)	Ref
Nausea			
Yes	23.8 (10/42)	20.4 (34/167)	1.23 (0.54–2.82)
No	76.2 (32/42)	79.6 (133/167)	Ref

^a^The information on self-reported symptoms were collected in questionnaires answered by the participants prior to examination and blood sample collection

^b^The participants were screened for celiac disease antibodies by EliA™ Celikey® tissue transglutaminase (TTG) anti-IgA and anti-IgG assays and deamidated gliadin peptide (DGP) anti-IgG assays. Celiac disease antibody positive were defined as IgG-DGP ≥ 10.0 U/mL and/or IgA-TTG/IgG-TTG ≥ 7.0 U/mL. 5 of the 16,776 participants had a diagnosis of celiac disease in the national patient register, they were all celiac disease antibody negative and were excluded before matching. Cases were matched without replacement on age (5 years range), sex and study to 4 controls

^c^Conditional logistic regression for comparison of cases with controls giving odds ratios (ORs) with 95% confidence intervals (CIs)

We compared levels of biomarkers between cases and controls, in Table 3 the odds ratios (ORs) are shown by quartiles. Only for the 1st quartile of cholesterol we found a significant association, indicating that individuals with low cholesterol are more likely to have undiagnosed celiac seropositivity. This is confirmed in the analyses with a clinical cut-off for cholesterol at 5 mmol/L, where we also found a significant association (OR 1.60, 95% CI 1.08–2.37). For hemoglobin we found no association when analyzing the quartiles, but when we use the clinical cut-off for anemia (7.3 mmol/L for women and 8.3 mmol/L for men) we found a significant association (OR 3.82, 95% CI 1.14–12.79), i.e. individuals with anemia are more likely to have undiagnosed celiac seropositivity. When we used the cut-off of 12 µg/L or less, used in an earlier study [9], we found a significant association of iron-deficiency and undiagnosed celiac seropositivity (OR:2.87, 95% CI 1.28–6.45, *p* = 0.011). We found no significant association for liver parameters and undiagnosed celiac seropositivity, nor for fasting blood sugar, serum 25-hydroxy Vitamin D or serum Vitamin B12.

**Table 3:** Comparison of levels of biomarkers between cases and controls

	N measurements (n cases/ n controls)	Cases: Celiac disease antibody positive participants^a^	Controls	OR (95% CI)^b^
Number of participants		169	676	
		Median (5%/95%)	Median (5%/95%)	
Cholesterol (mmol/L)*	790 (cases:158/controls:632)	5.5 (3.7/8.0)	5.7 (4.1/7.8)	
1st quartile				1.56 (0.92–2.62)
2nd quartile				1.02 (0.60–1.74)
3rd quartile				0.84 (0.50–1.43)
4th quartile				Ref
		% (n/N)	% (n/ total N )	
Cholesterol under 5 mmol/L		37.3 (59/158)	28.3 (179/632)	**1.60 (1.08–2.37)**
		Median (5%/95%)	Median (5%/95%)	
Hemoglobin (mmol/L)*	235 (cases:48/controls:187)	8.4 (7.2/9.8)	8.6 (7.6/9.9)	
1st quartile				1.44 (0.47–4.40)
2nd quartile				0.63 (0.21–1.83)
3rd quartile				0.63 (0.21–1.88)
4th quartile				Ref
		% (n/ total N )	% (n/N)	
Low hemoglobin^c^		12.5 (6/48)	4.3 (8/187)	**3.82 (1.14–12.79)**
		Median (5%/95%)	Median (5%/95%)	
Fasting blood glucose (mmol/L)*	549 (cases:110/controls:439)	4.8 (4.0/6.0)	4.8 (3.9/6.3)	
1st quartile				0.84 (0.46–1.54)
2nd quartile				1.00 (0.56–1.79)
3rd quartile				0.68 (0.38–1.23)
4th quartile				Ref
Fasting blood glucose over 7 mmol/L		1.8 (2/110)	2.5 (11/439)	0.69 (0.14–3.48)
		Median (5%/95%)	Median (5%/95%)	
HbA1c (%)*	474 (cases:95/controls:379)	5.8 (4.9/6.5)	5.8 (5.0/6.5)	
1st quartile				1.26 (0.61–2.57)
2nd quartile				1.01 (0.53–1.92)
3rd quartile				1.14 (0.62–2.08)
4th quartile				Ref
		Median (5%/95%)	Median (5%/95%)	
Ferritin (µg/L)*	455 (cases:92/controls:363)	74.5 (6.0/311.0)	78.0 (8.0/298.0)	
1st quartile				1.41 (0.65–3.05)
2nd quartile				0.72 (0.36–1.43)
3rd quartile				0.69 (0.35–1.34)
4th quartile				Ref
ALAT (Alanine transaminase)* (U/L)	465 (cases:94/controls:371)	10.8 (6.6/33.0)	10.2(6.6/32.0)	
1st quartile				0.67 (0.17–2.73)
2nd quartile				0.63 (0.15–2.56)
3rd quartile				1.43 (0.41–4.07)
4th quartile				Ref
ASAT (Aspartate aminotransferase) (U/L)*	35 (cases:7/controls:28)	20.0 (14.0/28.0)	17.5 (13.0/38.0)	
1st quartile				0.63 (0.08–5.26)
2nd quartile				0.37 (0.04–4.01)
3rd quartile				0.34 (0.03–4.41)
4th quartile				Ref
Alkaline phosphatase (ALP)* (U/L)	385 (cases:77/controls:308)	48.0 (28.2/90.0)	44.4 (28.2/72.0)	
1st quartile				0.69 (0.34–1.40)
2nd quartile				0.63 (0.32–1.25)
3rd quartile				0.60 (0.30–1.20)
4th quartile				Ref
		% (n/ total N )	% (n/ total N )	
Elevated liver parameters (ASAT/ALAT/ALP)^d^		1.0 (1/102)	2.2 (9/407)	0.42 (0.05–3.48)
		Median (5%/95%)	Median (5%/95%)	
Vitamin D (nmol/L)*	474 (cases:95/controls:379)	50.0 (10.0/107.0)	54.0 (13.0/102.0)	
1st quartile				1.21 (0.63–2.30)
2nd quartile				1.41 (0.76–2.65)
3rd quartile				1.05 (0.55–2.01)
4th quartile				Ref
Vitamin B12 (pmol/L)*	385 (cases:77/controls:308)	286.3 (159.4/513.6)	295.9 (170.5/492.2)	
1st quartile				1.22 (0.63–2.38)
2nd quartile				0.81 (0.39–1.69)
3rd quartile				0.64 (0.30–1.36)
4th quartile				Ref

*The quartiles were calculated per each of the eight studies

Statistically significant values are shown in bold

^a^The participants were screened for celiac disease antibodies by EliA™ Celikey® tissue transglutaminase (TTG) anti-IgA and anti-IgG assays and deamidated gliadin peptide (DGP) anti-IgG assays. Celiac disease antibody positive were defined as IgG-DGP ≥ 10.0 U/mL and/or IgA-TTG/IgG-TTG ≥ 7.0 U/mL. 5 of the 16,776 participants had a diagnosis of celiac disease in the national patient register, they were all celiac disease antibody negative and were excluded before matching. Cases were matched without replacement on age (5 years range), sex and study to 4 controls

^b^Conditional logistic regression for comparison of cases with controls giving odds ratios (ORs) with 95% confidence intervals (CIs)

^c^Low hemoglobin was defined as hemoglobin under 7.3 mmol/L for women and under 8.3 mmol/L for men

^d^Elevated liver parameters was defined as ALAT over 70 U/L for men and over 45 U/L for women, ASAT over 45 U/L for men and over 35 U/L for women, and alkaline phosphatase (ALP) over 105 U/L, 376 participants both had measurements for ALAT and ALP

## Discussion

In this study the 169 participants (1%) with undiagnosed celiac seropositivity were matched with four controls among the CD antibody negative participants. The cases and controls were compared regarding general symptoms, abdominal symptoms and biomarkers. Our results showed no significant differences in symptoms, but for low serum cholesterol measurements (< 5 mmol/L) and low blood hemoglobin (< 7.3 mmol/L for women and < 8.3 mmol/L for men) we found a significant association with undiagnosed celiac seropositivity. Thus, this study confirms the diagnostic difficulties of CD and the low prognostic value of symptoms for a diagnosis of CD.

This study confirm our previous findings [15] of no marked differences in symptoms among individuals with or without CD, also in this large population-based study comprising 16,771 individuals screened for CD antibodies. Hence, we also confirm the low predicative value of symptoms in the diagnostics of CD, found in other studies [6, 16, 17]. Furthermore, in line with others [31, 32] we found an association between decreased levels of cholesterol and undiagnosed celiac seropositivity. Also, in our earlier study we found that screening-detected clinical verified cases of CD had significantly lower cholesterol, but we found no significant association for the antibody positive participants in total. In agreement with anemia being a known complication to CD [1], and findings by West et al. [32], we found that low levels of blood hemoglobin had a significant association with undiagnosed celiac seropositivity, but we found no significant association in the quartiles-analyses. An American population-study [31] found no significant association between blood hemoglobin and undiagnosed CD, but they found a significant association for lower levels of serum ferritin. In the present study we found no significant differences in levels of serum ferritin among the cases and controls although there might be a tendency towards lower serum ferritin among the cases (74.5 vs. 78.0 µg/L). Also, Murray et al. [33] showed an association of iron-deficiency and CD. However, they used a cut-off for iron-deficiency of 12 µg/L or less. When we use the same cut-off in our study-population we find a significant association of iron-deficiency and undiagnosed celiac seropositivity (OR 2.87, 95% CI 1.28–6.45, *p* = 0.011). Indicating that for very low levels of serum ferritin, there might be an association with undiagnosed CD. It is however important to note the possibility of low iron, normal ferritin iron deficiency anemia, as ferritin is affected by other factors and can be in normal range fx. in patients with chronic disease.

In the present study we confirm the low prevalence of diagnosed CD; in this cohort 5 of the 16,776 screened individuals had a known diagnosis of CD in the National Patient Register (0.03%). This is consistent with a Danish register study showing the prevalence of diagnosed CD in adults increasing from 0.08% in 2006 to 0.18% in 2016 [13]. When we screen for CD antibodies in this population, we illustrate a large proportion of undiagnosed celiac seropositivity (0.03% vs. 1%), in line with our previous results [14, 15].

The population-based design is a strength of our study, since it may reflect a relevant non-clinical setting for screening of a wider population. Furthermore, the availability of stored blood samples for CD antibody measurements and linkage to nation-wide registries to exclude diagnosed cases of CD offered a unique opportunity to carry out this study. However, selection and response bias might be a limitation. Furthermore, our study might be limited by the slight uncertainty of the diagnosis of CD in the biobank analyses as clinical diagnosis and biopsies were not possible. Nevertheless, we know from our earlier study [15] that the positive predictive values of the chosen CD-antibody positivity were high. However, Hoerter et al. [34] found the positive predictive value of IgG DGP antibodies in TTG negative individuals to be low, this is a potential limitation, as 0.35% of this study population only were IgG-DGP positive. Additionally, even with such a large cohort there is a power problem as the prevalence of undiagnosed celiac seropositivity is 1%, and the number of cases therefore limited. It is also a limitation that we do not know the participants gluten intake, and there is a risk of misclassification of false negative if possible CD cases ate gluten-free-diet and consequently had a negative antibody screen-test. However, as these studies were conducted from 1976 to 2012 they were prior to the gluten-avoiding-trend recently described [35]. We also lack information on vitamin supplements, which could have affected the association of serum 25-hydroxy vitamin D and serum vitamin B12. The information on symptoms are collected from questionnaires, and there is a risk of recall bias and misclassification due to for example misunderstandings. Moreover, the differences in questionnaires and test for biomarkers, might be a limitation of the study, but as the cases were matched to the controls on study this limitation is limited. It is also likely that changes have occurred over time with regard to diagnostic criteria and development of improved antibody tests, which could influence our results. However, it is a strength of the study that serum samples from all eight cohorts were screened for CD antibodies with the same test: EliA™ Celikey® TTG anti-IgA and anti-IgG assay, and DGP anti-IgG assays. Another possible limitation is the potential degradation of immunoglobulins over time in frozen samples. An earlier study from our group investigated and confirmed the stability of IgE antibodies during eight years of storage in our biobank [36], but we do not have data on longer storage nor for other types of immunoglobulins.

## Conclusion

In this population-based study comprising 16,771 individuals 1% had undiagnosed celiac seropositivity, defined by CD antibody positivity with no known diagnosis of CD. Individuals with undiagnosed celiac seropositivity were compared with CD antibody negative matched controls, and we found no significant differences in general or abdominal symptoms, confirming the diagnostic difficulties of CD and the low prognostic value of symptoms for a diagnosis of CD. However, decreased levels of serum cholesterol as well as low blood hemoglobin was associated with undiagnosed celiac seropositivity.

## Supplementary Information

**Additional file 1.** Additional Material (Description of the population-based studies included), Additional Table 1 (Numbers of answers on symptoms for each symptom in the different cohorts), Additional Table 2 (Numbers and presence of biomarkers for each biomarker in the different cohorts) and Additional Table 3 (Questions from the questionnaires from the different cohorts translated to English).

## Data Availability

The data that support the findings of this study are available from Center for Clinical Research and Prevention and from national health registers but restrictions apply to the availability of these data, which were used under license for the current study, and so are not publicly available. Data are however available from the authors upon reasonable request and with permission from appropriate Danish authorities, as access to the data are subject to Danish regulations on personal data protection.

## Abbreviations

CD: Celiac disease
Ig: Immunoglobulin
TTG: Tissue transglutaminase
DGP: Deamidated gliadin peptide
ICD: International classification of disease
CI: Confidence interval
BMI: Body mass index
OR: Odds ratio
