# Supplementary material for: Symptoms and biomarkers associated with undiagnosed celiac seropositivity
Source: BMC Gastroenterol. 2021 Feb 27;21:90. doi: 10.1186/s12876-021-01667-y (PMC7913457; doi:10.1186/s12876-021-01667-y)
Supplement: Supplementary file 1 — Additional file 1. Additional Material (Description of the population-based studies included), Additional Table 1 (Numbers of answers on symptoms for each symptom in the different cohorts), Additional Table 2 (Numbers and presence of biomarkers for each biomarker in the different cohorts) and Additional Table 3 (Questions from the questionnaires from the different cohorts translated to English). [file 12876_2021_1667_MOESM1_ESM.docx]

**ADDITIONAL FILE 1**

**Symptoms and biomarkers associated with undiagnosed celiac seropositivity**

**Authors**: Line Lund Kårhus^1^, Janne Petersen^1,2^, Katja Biering Leth-Møller^1^, Line Tang Møllehave^1^, Anja Lykke Madsen^3^, Betina Heinsbæk Thuesen^1^, Peter Schwarz^4,5^, Jüri J. Rumessen^6^, Allan Linneberg^1,5^

*^1^ Center for Clinical Research and Prevention, Bispebjerg and Frederiksberg Hospital, Copenhagen, Denmark*

*^2^ Section of Biostatistics, Department of Public Health, University of Copenhagen, Denmark*

*^3^ The Copenhagen City Heart Study, Bispebjerg and Frederiksberg Hospital, Copenhagen, Denmark*

*^4^ Department of Endocrinology & Diabetes and Bone-metabolic Research Unit, Rigshospitalet, Copenhagen, Denmark*

*^5^ Department of Clinical Medicine, Faculty of Health and Medical Sciences, University of Copenhagen, Denmark*

*^6^ Q&D--Research Unit and Department of Gastroenterology, Herlev and Gentofte Hospital, University of Copenhagen, Denmark*

**Correspondence**:

Line Lund Kårhus, Center for Clinical Research and Prevention, Bispebjerg and Frederiksberg Hospital, Hovedvejen, Entrance 5, Nordre Fasanvej 57, DK-2000 Frederiksberg, Denmark.

E-mail: line.lund.kaarhus@regionh.dk Telephone: +45 38 16 31 23

**Contents**

[ADDITIONAL MATERIAL 3](#_Toc56665995)

[ADDITIONAL TABLES 7](#_Toc56666002)

[Additional Table 1: Numbers of answers on symptoms for each symptom in the different cohorts 7](#_Toc56666003)

[Additional Table 2: Numbers and presence of biomarkers for each biomarker in the different cohorts 7](#_Toc56666004)

[Additional Table 3: Questions from the questionnaires from the different cohorts translated to English 8](#_Toc56666005)

# ADDITIONAL MATERIAL

Description of the population-based studies included:

## *The 5-year follow-up of the Health2006 study*

The Health2006 study was a cross-sectional population-based study. A detailed description of the Health2006 cohort profile has been published elsewhere [1]. The participants invited to the baseline Health2006 study were drawn as a random sample from the background population aged 18–69 years, living in 11 municipalities in the western part of Copenhagen. All eligible participants in the Health2006 baseline study were invited to a 5-year follow-up examination including essentially the same study protocol [2,3] with the addition of screening for celiac disease by measurements of celiac disease biomarkers. The screening for celiac disease has been described elsewhere [3,4]. Brief, 2,297 individuals were screened for celiac disease antibodies by the Elia™ Celikey® tissue transglutaminase anti-IgA assay and deamidated gliadin peptide anti-IgA and anti-IgG assays, and screen-positive were referred to a gastroenterological exam including biopsies. For this present study only celiac disease antibody screen results were used.

## *The Inter99 Study*

The Inter99 study [5,6] was a population-based intervention study (CT00289237, ClinicalTrials.gov) investigating the effects of lifestyle intervention on cardiovascular disease (N=61,301) [6]. We used baseline data and blood sample drawn before participants were invited to the lifestyle intervention program. A random sample of 12,934 men and women aged between 30–60 years, living in 11 municipalities in the western part of Copenhagen, were invited to participate in a health examination during 1999–2001.

## *1936-cohort study (baseline examination at 40 years)*

The 1936-cohort study [7] is a longitudinal population-based study. A sample of 1,200 persons aged 40 years at the time of the study living in the western part of Copenhagen were invited to a health examination focused on cardiovascular risk factors. The participants completed a questionnaire on medical history, health and lifestyle. Between 1976 and 1977, a total of 1,052 persons were examined [7,8].

##

## *The Monica studies*

The MONICA studies was a part of an international World Health Organization (WHO) co-ordinated study, MONItoring of trends and determinants in CArdiovascular Diseases [9]. Between 1982 and 1995 three repeated cross-sectional studies (MONICA I, II, and III) were carried out, inviting random samples of equal numbers of men and women in the age groups 30, 40, 50 and 60 years, and living in the western part of Copenhagen, drawn from the Danish Civil Registration System (in which all inhabitants in Denmark are registered) [10].

*The Monica-I study*

The Monica-I study [10] took place from 1982 to 1984. For the Monica-I study 4,807 individuals were invited and 3,785 participated. For this present study only blood samples from 1,839 women were included and screened for celiac disease antibodies, because there was no remaining serum in the biobank from male participants in the study.

*The Monica-II study*

The Monica-II study [11] took place from 1986 to 1987. For the Monica-II study 2,000 individuals were invited and 1,504 participated.

*The Monica-III study*

The Monica-III study [10,11] took place from 1991 to 1992. For the Monica-III study 2,927 individuals were invited and 2,027 participated.

## *The 1914-cohort study (examination at 70 years)*

The 1914-cohort study [12,13] is a longitudinal study of health and aging, initially aimed to investigate risk factors for heart diseases, but the purpose was widened in 1984 to also include the aging process. In 1964, a total of 976 individuals born in 1914 and living in seven municipalities in the western part of Copenhagen were invited, and 802 (436 men and 366 women) participated. Repeated assessments occurred at 10-year intervals from age 50, and every 5 years from age 75. For the examination at 70 and 75 years of age, the sample was refreshed in order to increase the sample size, here individuals born in 1914 were drawn from the National Person Register. For this study we use the blood samples from the examination at age 70 years of age where data was obtained from two groups of individuals born in 1914 and aged 70 years: 1) follow-up from the baseline of the 1914-cohort started in 1964, where 726 individuals were eligible for invitation and 537 participated (73%). 2) The additional 383 individuals invited to supplement the cohort, where 267 participated (70%). Thus, in total the study population for the 1914-cohort study examination at 70 years was 804 individuals [12,13].

##

## *The Allergy90 study*

The Allergy90 study [14,15], also known as The Copenhagen Allergy study, was a cross-sectional study conducted in 1990. In a first phase, a screening questionnaire of respiratory symptoms, was mailed to a random sample of 15- to 69- year old individuals living in 11 municipalities in the western part of Copenhagen. 6,998 individuals responded to the questionnaire (86 %). A random group of 793 individuals and a symptom group of 788 individuals were selected among the respondents and invited to a health examination. 599 (76%) in the random group and 635 (81%) in the symptom group were examined (122 individuals were in both groups). Thus, 1,112 individuals were examined in total. The examinations were conducted from February 1990 until January 1991 [14,15].

**REFERENCES**

1 Thuesen BH, Cerqueira C, Aadahl M, Ebstrup JF, Toft U, Thyssen JP, et al. Cohort Profile: the Health2006 cohort, research centre for prevention and health. Int J Epidemiol 2014;43:568-75.

2 Gonzalez-Quintela A, Dam Laursen AS, Vidal C, Skaaby T, Gude F, Linneberg A. IgE antibodies to alpha-gal in the general adult population: relationship with tick bites, atopy, and cat ownership. Clin Exp Allergy 2014;44:1061-8.

3 Horwitz A, Skaaby T, Kårhus LL, Schwarz P, Jorgensen T, Rumessen JJ, et al. Screening for celiac disease in Danish adults. Scand J Gastroenterol 2015;50:824-31.

4 Kårhus LL, Thuesen BH, Rumessen JJ, Linneberg A. Symptoms and biomarkers associated with celiac disease: evaluation of a population-based screening program in adults. Eur J Gastroenterol Hepatol 2016;28:1298-304.

5 Jorgensen T, Borch-Johnsen K, Thomsen TF, Ibsen H, Glumer C, Pisinger C. A randomized non-pharmacological intervention study for prevention of ischaemic heart disease: baseline results Inter99. Eur J Cardiovasc Prev Rehabil 2003;10:377-86.

6 Jorgensen T, Jacobsen RK, Toft U, Aadahl M, Glumer C, Pisinger C. Effect of screening and lifestyle counselling on incidence of ischaemic heart disease in general population: Inter99 randomised trial. BMJ 2014;348:g3617.

7 Drivsholm T, Eplov LF, Davidsen M, Jorgensen T, Ibsen H, Hollnagel H, et al. Representativeness in population-based studies: a detailed description of non-response in a Danish cohort study. Scand J Public Health 2006;34:623-31.

8 Linneberg A, Friedrich N, Husemoen LL, Thuesen B, Gonzalez-Quintela A, Vidal C, et al. Incidence and remission of specific IgE aeroallergen sensitization from age of 40 to 60 years, and association with alcohol consumption. Int Arch Allergy Immunol 2010;151:142-8.

9 The World Health Organization MONICA Project (monitoring trends and determinants in cardiovascular disease): a major international collaboration. WHO MONICA Project Principal Investigators. J Clin Epidemiol 1988;41:105-14.

10 Gerdes LU, Bronnum-Hansen H, Madsen M, Borch-Johnsen K, Jorgensen T, Sjol A, et al. Trends in selected biological risk factors for cardiovascular diseases in the Danish MONICA population, 1982-1992. J Clin Epidemiol 2000;53:427-34.

11 Sjol A, Thomsen KK, Schroll M. Secular trends in blood pressure levels in Denmark 1964-1991. Int J Epidemiol 1998;27:614-22.

12 Avlund K, Kreiner S, Schultz-Larsen K. Construct validation and the Rasch model: functional ability of healthy elderly people. Scand J Soc Med 1993;21:233-46.

13 Stovring N, Avlund K, Schultz-Larsen K, Schroll M. The cumulative effect of smoking at age 50, 60, and 70 on functional ability at age 75. Scand J Public Health 2004;32:296-302.

14 Linneberg A, Husemoen LL, Nielsen NH, Madsen F, Frolund L, Johansen N. Screening for allergic respiratory disease in the general population with the ADVIA Centaur Allergy Screen Assay. Allergy 2006;61:344-8.

15 Linneberg A, Nielsen NH, Madsen F, Frolund L, Dirksen A, Jorgensen T. Smoking and the development of allergic sensitization to aeroallergens in adults: a prospective population-based study. The Copenhagen Allergy Study. Allergy 2001;56:328-32.

# ADDITIONAL TABLES

## Additional Table 1: Numbers of answers on symptoms for each symptom in the different cohorts

|  | **Total N** | **Headache** | **Self-perceived health** | **Tired** | **Bloating** | **Reflux** | **Rumbling** | **Abdominal pain** | **Nausea** | **Alternating stool** |
| --- | --- | --- | --- | --- | --- | --- | --- | --- | --- | --- |
| **Allergy90** | 55 | 55 | 55 | 55 |  |  |  | 55 |  |  |
| **Health2006** | 90 | 90 | 90 | 90 | 90 | 90 | 90 | 90 | 90 | 90 |
| **Inter99** | 385 | 379 | 382 | 378 | 378 |  | 377 | 379 |  | 381 |
| **1914 cohort** | 35 | 35 | 35 | 35 | 35 | 35 | 35 | 35 | 35 | 35 |
| **1936 cohort** | 40 | 40 | 40 | 40 |  |  | 40 | 40 |  | 40 |
| **Monica I** | 85 | 84 |  | 85 | 84 | 84 | 84 | 85 | 84 | 82 |
| **Monica II** | 60 |  | 60 | 59 |  | 56 | 59 | 57 |  | 56 |
| **Monica III** | 95 | 81 | 75 | 95 |  | 92 | 94 | 93 |  | 95 |
| **Total N** | **845** | **764** | **737** | **837** | **587** | **357** | **779** | **834** | **209** | **779** |

## Additional Table 2: Numbers and presence of biomarkers for each biomarker in the different cohorts

|  | **Total N** | **Cholesterol** | **Hemoglobin** | **Fasting blood sugar** | **Vit. D** | **HbA1c** | **ALAT** | **ASAT** | **ALP** | **Ferritin** | **Vit. B12** |
| --- | --- | --- | --- | --- | --- | --- | --- | --- | --- | --- | --- |
| **Allergy90** | 55 |  |  |  |  |  |  |  |  |  |  |
| **Health2006** | 90 | 90 | 90 | 90 | 90 | 90 | 89 |  |  |  |  |
| **Inter99** | 385 | 385 |  | 385 | 384 | 384 | 376 |  | 385 | 385 | 385 |
| **1914 cohort** | 35 | 35 | 35 | 34 |  |  |  | 35 |  |  |  |
| **1936 cohort** | 40 | 40 | 40 | 40 |  |  |  |  |  |  |  |
| **Monica I** | 85 | 85 | 70 |  |  |  |  |  |  | 70 |  |
| **Monica II** | 60 | 60 |  |  |  |  |  |  |  |  |  |
| **Monica III** | 95 | 95 |  |  |  |  |  |  |  |  |  |
| **Total N** | **845** | **790** | **235** | **549** | **474** | **474** | **465** | **35** | **385** | **455** | **385** |

## Additional Table 3: Questions from the questionnaires from the different cohorts translated to English

| **Tiredness** | | |
| --- | --- | --- |
|  | **Questions** | **Answers** |
| 1914 cohort | Do you feel tired? | Yes/no |
| 1936 cohort | Do you feel tired? | Yes/no |
| Monica I | Do you often feel tired? | Yes/no |
| Monica II | Do you feel tired? | Yes/no |
| Monica III | Do you often feel tired? | Yes/no |
| Allergy90 | Do you often feel very tired? | Yes/no |
| Inter99 | Do you often feel very tired? | Yes/no |
| Health2006 | Do you often feel very tired? | Yes/no |
| **Self-perceived health** | | |
|  | **Questions** | **Answers** |
| 1914 cohort | How would you report your health during the last year? | Very good, Good, Fair, Poor |
| 1936 cohort | How would you report your health during the last year? | Very good, Good, Fair, Poor |
| Monica I | How would you report your health at the moment? | Excellent, Very good, Good,  Fair,Poor |
| Monica II | How would you report your health at the moment? | Excellent, Very good, Good,  Fair,Poor |
| Monica III | How would you report your health at the moment? | Excellent, Very good, Good,  Fair,Poor |
| Allergy90 | How would you report your health the last 12 months? | Very good, Good, Fair,Poor |
| Inter99 | How would you report your health in total? | Excellent, Very good, Good,  Fair,Poor |
| Health2006 | How would you report your health in total? | Excellent, Very good, Good,  Fair,Poor |
| **Headache** | | |
|  | **Questions** | **Answers** |
| 1914 cohort | Do you often have a headache? | No, never  Yes, a couple of times per year  Yes, a couple of times per month  Yes, a couple of times per week  Yes, constantly |
| 1936 cohort | Do you often have a headache? | No, never  Yes, a couple of times per year  Yes, a couple of times per month  Yes, a couple of times per week  Yes, constantly |
| Monica I | Do you often have a strong headache? | Yes/no |
| Monica III | Do you often have a strong headache? | Yes/no |
| Allergy90 | Do you often have a strong headache? | No, never  Yes, a couple of times per year  Yes, a couple of times per month  Yes, a couple of times per week  Yes, constantly |
| Inter99 | Do you often have a headache? | Yes/no |
| Health2006 | Do you often have a strong headache? | Yes/no |

| **Abdominal pain** | | |
| --- | --- | --- |
|  | **Questions** | **Answers** |
| 1914 cohort | Have you had abdominal pains the last 12 months? | Yes/No |
| 1936 cohort | Do you suffer from abdominal pain? | Yes/No |
| Monica I | Have you had abdominal pains the last 12 months? | Yes, often  Yes, sometimes  No |
| Monica II | Do you suffer from abdominal pain? | Yes, almost constantly  Yes, often  Yes, sometimes  No, never |
| Monica III | Do you suffer from abdominal pain? | Yes, almost constantly  Yes, often  Yes, sometimes  No, never |
| Allergy90 | Have you had abdominal pains the last 12 months? | Yes, often  Yes, sometimes  No |
| Inter99 | Do you suffer from abdominal pain? | Yes, almost daily  Yes, weekly  Yes, monthly  Seldom or never |
| Health2006 | Do you suffer from abdominal pain? | Yes, almost daily  Yes, weekly  Yes, monthly  Seldom or never |
| **Abdominal symptoms** | | |
|  | **Questions** | **Answers** |
| 1914 cohort | Do you have alternating stools?  Do you suffer from nausea?  Do you suffer from rumbling in the stomach?  Do you suffer from heartburn?  Do you feel bloated? | Yes, almost constantly  Yes, often  Yes, sometimes  No, never |
| 1936 cohort | Do you have alternating stools?  Do you suffer from rumbling in the stomach? | Yes, almost constantly  Yes, often  Yes, sometimes  No, never |
| Monica I | Do you have alternating stools?  Do you suffer from nausea?  Do you suffer from rumbling in the stomach?  Do you suffer from heartburn?  Do you feel bloated? | Yes, almost constantly  Yes, often  Yes, sometimes  No, never |
| Monica II | Do you have alternating stools?  Do you suffer from rumbling in the stomach?  Do you suffer from heartburn? | Yes, almost constantly  Yes, often  Yes, sometimes  No, never |
| Monica III | Do you have alternating stools?  Do you suffer from rumbling in the stomach?  Do you suffer from heartburn? | Yes, almost constantly  Yes, often  Yes, sometimes  No, never |
| Inter99 | Do you have alternating stools?  Do you suffer from rumbling in the stomach?  Do you feel bloated? | Yes, almost constantly  Yes, often  Yes, sometimes  No, never |
| Health2006 | Do you have alternating stools?  Do you suffer from nausea?  Do you suffer from rumbling in the stomach?  Do you suffer from heartburn?  Do you feel bloated? | Yes, almost constantly  Yes, often  Yes, sometimes  No, never |
